# Identification of genes related to fatty acid metabolism in type 2 diabetes mellitus

**DOI:** 10.1016/j.bbrep.2024.101849

**Published:** 2024-10-22

**Authors:** Ji Yang, Yikun Zhou, Jiarui Zhang, Yongqin Zheng, Jundong He

**Affiliations:** a Medical School, Kunming University of Science and Technology, Kunming, Yunnan, China; b Department of Endocrinology and Metabolism, The First People's Hospital of Yunnan Province, The Affiliated Hospital of Kunming University of Science and Technology, Kunming, Yunnan, China

**Keywords:** Type 2 diabetes mellitus, Fatty acid metabolism, Biomarkers for T2DM

## Abstract

**Aim:**

Fatty acid metabolism is pivotal for lipid synthesis, cellular signaling, and maintaining cell membrane integrity. However, its diagnostic significance in type 2 diabetes mellitus (T2DM) remains unclear.

**Materials and methods:**

Three datasets and fatty acid metabolism-related genes were retrieved. Differential expression analysis, WGCNA, machine learning algorithms, diagnostic analysis, and validation were employed to identify key feature genes. Functional analysis, ceRNA network construction, immune microenvironment assessment, and drug prediction were conducted to explore the underlying molecular mechanisms.

**Results:**

Six feature genes were identified with strong diagnostic performance and were involved in processes such as ribosome function and fatty acid metabolism. Immune cells, including dendritic cells, eosinophils, and neutrophils, may play a role in the progression of T2DM. ceRNA and drug-target network analysis revealed potential interactions, such as RP11-miR-29a-YTHDF3 and BPA-MSANTD1. The expression patterns of the feature genes, except for YTHDF3, were consistently upregulated in T2DM, aligning with trends observed in the training set.

**Conclusion:**

This study investigated the potential molecular mechanisms of six fatty acid metabolism-related genes in T2DM, offering valuable insights that may guide future research and therapeutic development.

## Highlights

• Six fatty acid metabolism-related genes for T2DM diagnosis were identified.
• Correlations between the feature genes and differential immune cells were demonstrated.
• The construction of ceRNA and drug-target networks provided a new perspective for the treatment of T2DM.

## 1 Introduction

Diabetes mellitus (DM) poses a significant global health challenge, with its prevalence steadily increasing. In 2021, 537 million individuals were affected by DM, and projections estimate this number will rise to 783 million by 2045 [1]. Dysregulation of glucose metabolism has detrimental effects on blood vessel integrity, leading to multiple organ dysfunction. Diabetes-related complications manifest as either macrovascular (e.g., cardiomyopathy, arteriopathy) or microvascular (e.g., nephropathy, retinopathy, neuropathy) disorders. Notably, over 90 % of diabetes cases are classified as type 2 DM (T2DM), with its incidence alarmingly increasing among younger populations, including children and adults under 40 years of age [2]. T2DM is a chronic condition characterized by insulin secretion deficiency, beta-cell dysfunction, and insulin resistance. Strikingly, 30 %–50 % of T2DM cases remain undiagnosed and untreated [3,4]. Early identification of patients is critical for mitigating microvascular and macrovascular complications, as well as reducing mortality rates. Consequently, the development of personalized and effective molecular diagnostic markers is urgently needed for the treatment and management of diverse T2DM cases.

At the core of lipid metabolism lies the intricate process of fatty acid metabolism, which plays a fundamental role in regulating cellular bioenergetic homeostasis in mammalian cells. Fatty acids undergo rapid oxidation within peroxisomes and mitochondria, producing significant energy in the form of adenosine triphosphate (ATP) when sufficient oxygen is present [5]. This highly efficient energy production process is essential for maintaining various cellular functions. However, abnormalities in fatty acid oxidation have been strongly associated with the pathogenesis of T2DM and obesity. Disruption of the delicate balance of fatty acid metabolism can contribute to insulin resistance and metabolic dysregulation, ultimately exacerbating the progression of T2DM and obesity [6,7]. Therefore, the identification of key genes related to fatty acid metabolism provides novel insights into the management and mitigation of T2DM.

This study aimed to evaluate the diagnostic potential of fatty acid metabolism-related genes (FMGs) in T2DM. T2DM-related datasets were obtained from the Gene Expression Omnibus (GEO) database, and FMGs were extracted from the Molecular Signatures Database (MSigDB). Key feature genes associated with T2DM were identified using a combination of differential expression analysis, weighted gene co-expression network analysis (WGCNA), and machine learning techniques. Additionally, these feature genes were analyzed through gene set enrichment analysis (GSEA) and immune infiltration analysis to elucidate their role in T2DM pathogenesis, providing valuable insights for disease management. The results of this investigation aim to lay the groundwork for understanding the involvement of fatty acid metabolism in T2DM and offer novel targets for therapeutic interventions.

## 2 Materials and methods

### 2.1 Data source

**Fig. 1:**
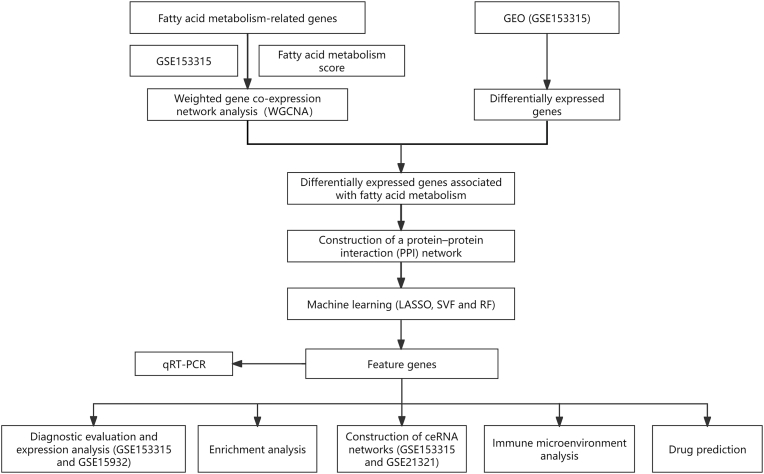
Flowchart for the identification of genes involved in fatty acid metabolism in T2DM.

The flowchart of this study was shown in Fig. 1. In this study, we obtained T2DM-related datasets GSE153315, GSE21321, and GSE15932 from the GEO database (https://www.ncbi.nlm.nih.gov/gds). GSE153315 included mRNA and long non-coding RNA (lncRNA) data (10 normal blood samples, 20 T2DM blood samples) sequenced using the GPL17303 Ion Torrent Proton platform. GSE21321 provided microRNA (miRNA) data (10 normal blood samples, 9 T2DM blood samples), while GSE15932 was used for biomarker expression validation and diagnostic performance analysis (8 normal blood samples, 8 T2DM blood samples). Additionally, we compiled age, gender, and other relevant subject information for the three datasets in Table 1. Three sets of FMGs were retrieved from the MSigDB database (https://ngdc.cncb.ac.cn/databasecommons) [8], yielding a total of 309 fatty acid metabolism-related genes (FMGs) after deduplication and merging.

**Table 1:** The information of subjects in three datasets.

GEO ID	Platform	RNA	Organism	Samples (Case/Control)	Tissue	Age (mean)	Gender (male/female)	Country	Drug
GSE153315	GPL17303	mRNA lncRNA	Homo sapiens	20/10	Whole blood	49.1	13/17	US	Metformin
GSE15932	GPL570	mRNA	Homo sapiens	8/8	Peripheral blood	69.375	10/6	UK	–
GSE21321	GPL10322	miRNA	Homo sapiens	9/10	Blood	–	19/0	Singapore	–

Notes: "-" indicates unknown information.

### 2.2 Acquisition of differentially expressed genes (DEGs)

To identify DEGs between sample groups and facilitate further exploration of these genes, we analyzed the DEGs between the T2DM group and the normal group in the training set GSE153315 using the limma package (v 3.42.2) [9]. The screening criteria were set at *P* < 0.05 and |log_2_(fold change)| > 0.5 between normal and T2DM samples. The results were visualized using a volcano plot and a heatmap of the DEGs.

### 2.3 Weighted gene co-expression network analysis (WGCNA)

To identify genes associated with fatty acid metabolism, we first calculated the fatty acid metabolism (FAM) score for samples in the GSE153315 dataset using single-sample Gene Set Enrichment Analysis (ssGSEA) from the GSVA package (v 1.42.0) [10]. Next, WGCNA was performed using the FAM score as a trait with the WGCNA package (v 1.70.3). WGCNA is an analytical method for analysing gene expression patterns in multiple samples to analyze associations between modules and specific traits or phenotypes. Genes with a covariance exceeding 25 % were selected, and outliers were removed through sample clustering. To ensure that inter-gene interactions were maximally consistent with a scale-free distribution, a soft threshold was determined to construct the network. The dissimilarity coefficient, adjacency, and similarity among genes were computed to identify modules containing more than 30 genes based on dynamic tree-cutting criteria. These modules were then correlated with clinical features, and the results were visualized in a heatmap. The most positively and negatively correlated modules with FAM scores were identified, along with hub genes (*P* < 0.05 and |cor| > 0.7). Differentially expressed FMGs (DE-FMGs) were obtained by intersecting the DEGs and hub genes using the online tool jVenn.

### 2.4 Construction of a protein-protein interaction (PPI) network

To explore potential interactions among the DE-FMGs, we analyzed their interactions using the STRING website (http://string-db.org) with a confidence threshold of 0.4). The MCODE plug-in was then employed to analyze the PPI network, identifying key submodules within the network through specific filtering criteria. With the MCODE plug-in, PPI networks could be analyzed in depth, filtering out noise and minor connections. The resulting network graph was visualized using Cytoscape software.

### 2.5 Screening for feature genes

Candidate genes were obtained through univariate logistic regression analysis on DE-FMGs from GSE153315 (*P* < 0.05) and presented in a forest plot. The least absolute shrinkage and selection operator (LASSO) analysis was applied using the parameters family = "binary", type.measure = "class", and nfold = 10 with the glmnet package (v 4.0-2) [11]. LASSO analysis is the compression and selection of feature coefficients by adding an L1 regularisation term to the loss function of a linear regression model, which reduces computational costs and improves the model's interpretability, predictive performance and generalisation. Support vector machines (SVMs) were implemented using the e1071 package (v 1.7–9) to rank candidate genes, with recursive feature elimination (RFE) used to determine their importance ranking [12]. The SVM algorithm is designed to separate two types of data by finding an optimal hyperplane, which is able to handle high-dimensional data, avoid overfitting phenomenon and reduce generalisation error. Random forest (RF) analysis was performed using the randomForest package (v 3.36.0) [13], and genes were ranked based on the "Mean Decrease Accuracy" and "Mean Decrease Gini" methods. RF constructs predictive models by sampling objects and variables, generates multiple decision trees, and classifies objects sequentially, it can be able to efficiently handle datasets with high-dimensional features (multivariate) and does not require dimensionality reduction. Feature genes were identified by intersecting the genes selected through LASSO, SVM, and RF analyses.

### 2.6 Diagnostic evaluation and expression analysis

In the GSE153315 dataset, the discriminatory potential of each feature gene in distinguishing T2DM from normal samples was evaluated by plotting receiver operating characteristic (ROC) curves using the pROC package (v 1.12.1) [14]. A parallel ROC curve analysis was performed on the GSE15932 dataset to validate the diagnostic capability of the feature genes. Additionally, the expression levels of the feature genes were extracted from both GSE153315 and GSE15932 and compared between normal and T2DM samples. The results were visualized using box plots.

### 2.7 Enrichment analysis of characterized genes

To explore the functional pathways associated with the feature genes, first, we calculated the pearson correlation coefficients between the feature genes and all genes separately, ranked the correlation coefficients from the largest to the smallest, and then Gene Ontology (GO) and Kyoto Encyclopedia of Genes and Genomes (KEGG) analyses were conducted using the clusterProfiler package (v 3.14.3) [15]. In addition, Benjamini & Hochberg methods were used for multiple test correction, *P*.adjust<0.05 was considered as significant enrichment. The top five KEGG and GO pathways were selected for presentation.

### 2.8 Screening for differentially expressed lncRNAs (DE-lncRNAs) and miRNAs

Differentially expressed lncRNAs (DE-lncRNAs) and miRNAs (DE-miRNAs) between T2DM and normal samples in GSE153315 and GSE21321 datasets were respectively identified using the limma package [9], with a threshold of *P* < 0.05 and |log2(fold change)| > 0.5. The results were visualized using volcano plots and heat maps.

### 2.9 Construction of a competing endogenous RNA (ceRNA) network

Competing endogenous RNA (ceRNA) functions as a competitive binding element for RNA. Initially, the miRWalk 3.0 database was used to predict miRNAs associated with feature genes (mRNAs) with a binding probability ≥0.95 and binding site position specified as “3UTR”). Similarly, miRNA prediction was performed using the ENCORI database with the following thresholds: CLIP-Data ≥1, Degradome-Data ≥0, pan-Cancer ≥0, and programNum ≥1. The results obtained from the two prediction methods were deduplicated and merged. Predicted miRNAs were then intersected with DE-miRNAs to obtain mRNA–miRNA pairs. Subsequently, lncRNA prediction was conducted for these pairs using LncBase v2.0 with a threshold score >0.9. Predicted lncRNAs were intersected with DE-lncRNAs to generate miRNA–lncRNA pairs. Finally, mRNA–miRNAs and miRNA–lncRNAs pairs were overlapped to identify lncRNA–miRNA–mRNA relationship trios. A ceRNA network diagram was constructed to visualize the results.

### 2.10 Immune microenvironment analysis

The ssGSEA algorithm can assess the relative enrichment of a gene set in a single sample by calculating its enrichment score. Thus, it was used to calculate the 28 immune cell infiltration abundance for all samples in the GSE153315 dataset. Correlations between feature genes and significantly different immune cells were then calculated using the Spearman method. A box plot, generated using the ggplot2 package (v 3.3.2) was used to display the differential expressions of immune cells between T2DM and normal samples. Additionally, a lollipop plot was created to visualize the correlations between feature genes and differential immune cells.

### 2.11 Drug prediction of feature genes based on in-silico analysis

Drugs associated with T2DM were identified using feature genes as keywords in the Comparative Toxicogenomics Database. The results were visualized as a drug–target network diagram, constructed using Cytoscape software.

### 2.12 Expression of characterised genes was verified by wet-lab experiments using Quantitative real-time polymerase chain reaction (qRT-PCR)

Ten pairs of frozen human peripheral blood samples were collected from The First People's Hospital of Yunnan Province, classified into T2DM (N = 10) and normal (N = 10) groups. Informed consent was obtained from all participants, and the study was approved by the ethics committee of The First People's Hospital of Yunnan Province (NO: KHLL2021-KY012). Total RNA was extracted from 500 μL blood using TRIzol reagent, and RNA concentration was measured using 1 μL of RNA with the NanoPhotometer N50. The RNA was reverse-transcribed into cDNA using the SureScript First-Strand cDNA Synthesis Kit (Servicebio) cDNA, then diluted 5–20 times with RNase/DNase-free double-distilled water and subjected to qRT-PCR. The following protocol was used: pre-denaturation at 95 °C for 1 min, denaturation at 95 °C for 20 s, annealing at 55 °C for 20 s, and extension at 72 °C for 30 s, repeated for 40 cycles. Glyceraldehyde 3-phosphate dehydrogenase was used as the internal reference for gene expression detection. Finally, the expression levels of the feature genes were compared between T2DM and normal groups. Primer sequences are provided in Supplementary Table 1.

## 3 Results

### 3.1 Differential expression analysis between T2DM and normal sample groups

**Fig. 2:**
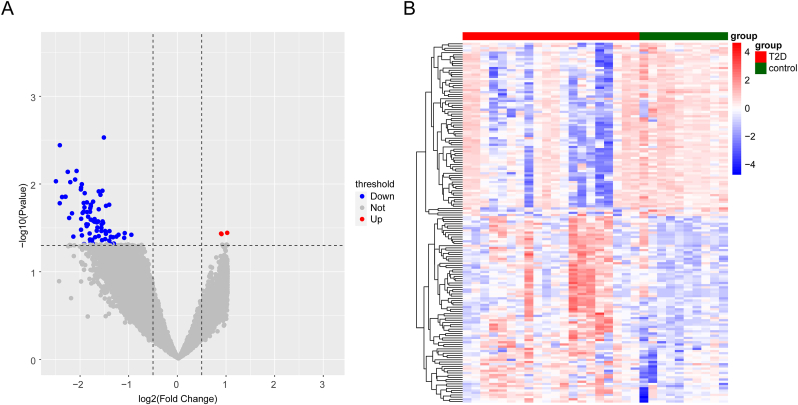
Differential expression of genes (DEGs) between type 2 diabetes mellitus (T2DM) samples and normal samples. T2D in the diagram represents T2DM. (A) Volcano plots of genes that differ between sample groups. Red indicated up-regulation, blue indicated down-regulation. (B) Heatmap of expression of differential genes between sample groups. Red was high expression and blue was low expression.

A total of 164 DEGs were identified between T2DM and normal samples, including 85 upregulated and 79 downregulated DEGs. The distribution of DEGs was visualized using a volcano plot, while their expression differences between T2DM and normal samples were illustrated in a heat map (Fig. 2A and. B).

### 3.2 Identification of DE-FMGs

**Fig. 3:**
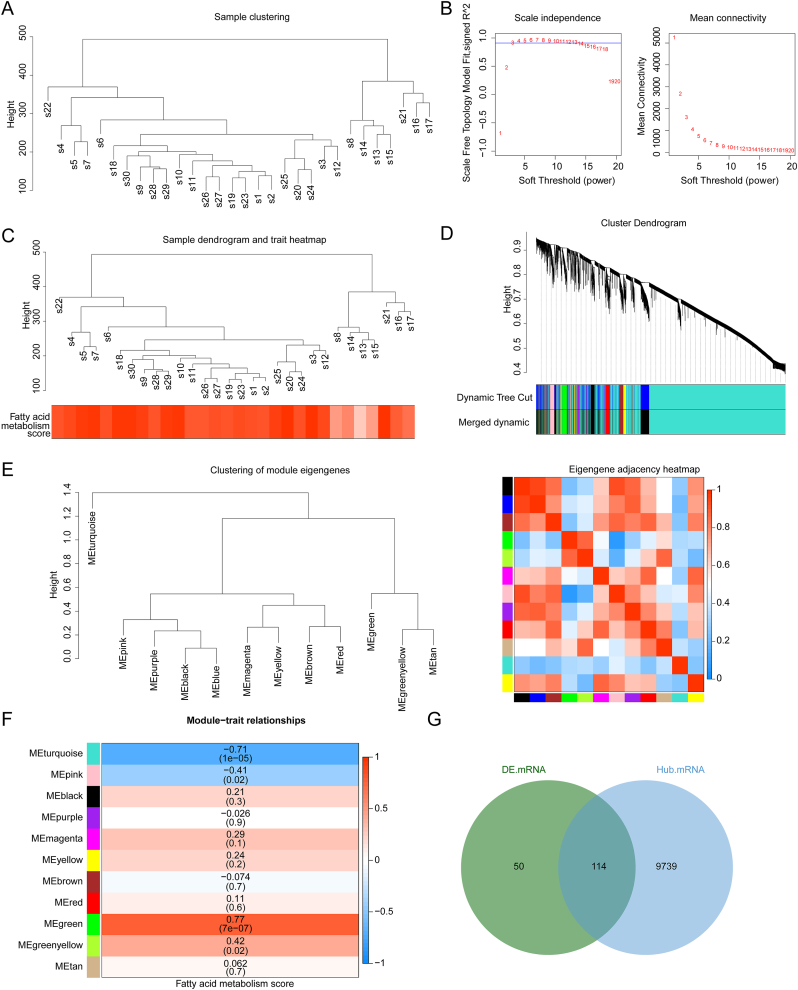
Identification of hub genes and differentially expressed feature genes (DE-FMGs). (A) Clustering of dataset samples. (B) Scale-free soft threshold distribution. (C) Clustering of data samples and dendrogram of phenotypic information. (D) Tree diagram illustrating module clustering. Different colors represent distinct clustering modules. (E) Clustering of module eigengenes and eigengene adjacency heatmap. (F) Relationships between module genes and fatty acid metabolism score. (G) Intersection of hub genes and DEGs.

Among the 13,401 genes with variance exceeding 25 %, the overall clustering of dataset samples indicated that no sample removal was necessary (Fig. 3A and. B). In the WGCNA analysis, a power value of 3 was selected at optimal, based on the position of the blue line in Fig. 3C, suggesting a network that approaches scale-free distribution. A total of 11 modules were identified through the construction of co-expression matrices (Fig. 3D and. E). The green and turquoise modules were the most positively and negatively correlated with the FAM score, containing 302 and 9551 genes, respectively. Combining these two key modules resulted in 9853 hub genes (Fig. 3F). The intersection of hub genes and DEGs yielded 114 DE-FMGs (Fig. 3G).

### 3.3 The KDELR3-KLC3 and RHOU-SOX4 key submodules

**Fig. 4:**
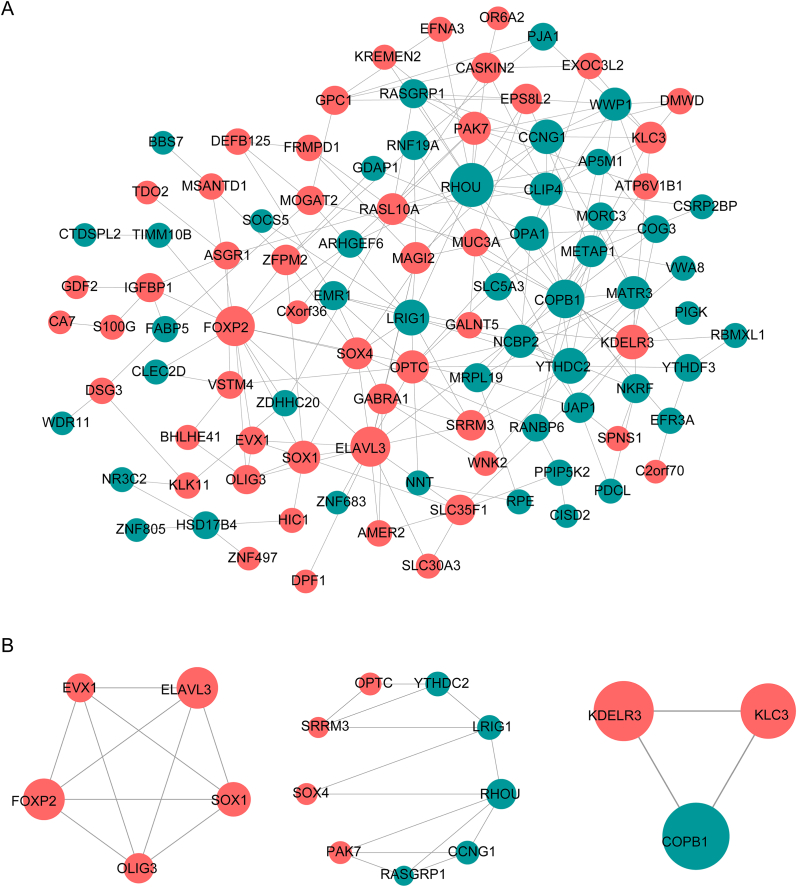
Protein-protein interaction (PPI) network. (A) PPI network depicting interactions among the 114 DE-FMGs. (B) PPI networks highlighting three key submodules.

The PPI network consisted of 212 protein interaction pairs involving 98 nodes, including notable interactions such as FOXP2–CLEC2D and ELAVL3–SOX1 (Fig. 4A). Three key submodules were identified, including KDELR3–KLC3 and RHOU–SOX4 (Fig. 4B).

### 3.4 Identification of feature genes

**Fig. 5:**
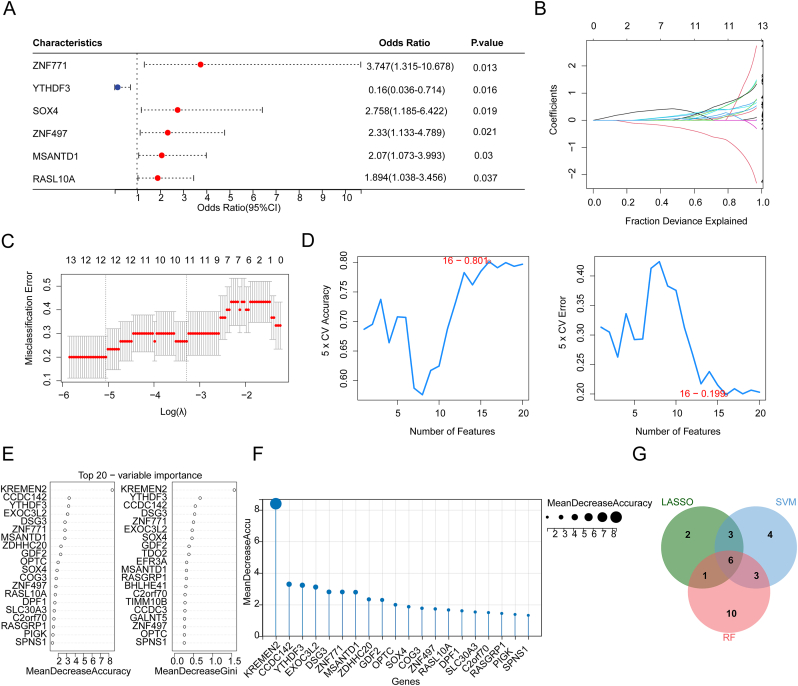
Screening for feature genes. (A) Forest plot presenting the six candidate genes. (B) Plot of candidate genes coefficient changes in the least absolute shrinkage and selection operator (LASSO) model. (C) LASSO logic coefficient penalty plot. As the penalty coefficients lambda were varied, the coefficients were eventually compressed to 0 for most variables, and the best lambda value was selected at the minimum of the 10-fold cross-validation error. (D) Importance ranking of candidate genes obtained through Support vector machines (SVMs) algorithm and recursive feature elimination (RFE) methods. The best combination, determined by accuracy rate and lowest error rate, yielded the corresponding gene. SVMs model accuracy (left) and error rate (right) (E, F) Analysis of the top 20 important genes using the RF method, ranked by "Mean Decrease Accuracy" and "Mean Decrease Gini" methods. (G) Intersection of feature genes identified through LASSO, SVM, and random forest (RF).

Univariate logistic regression analysis identified 46 candidate genes significantly associated with T2DM, with a forest plot depicting six of them (Fig. 5A). At the optimal lambda value (lambda.min = 0.00638129), the lowest error rate was achieved, resulting in 12 genes being selected: CA7, CCDC3, EXOC3L2, KDELR3, KLK11, MATR3, MSANTD1, RASL10A, SOX4, YTHDF3, ZNF497, and ZNF771 (Fig. 5B and. C). Based on SVM algorithm, we calculated the accuracy of the model for different feature combinations using 5-fold cross-validation, and the model reached its highest accuracy after incorporating the first 16 features. The 16 feature genes were screened: RASL10A, SOX4, YTHDF3, ZNF497, ZNF771, SPNS1, KLK11, CXorf36, MATR3, KDELR3, EFNA3, RASGRP1, OR12D3, RNF19A, KREMEN2, and MSANTD1 (Fig. 5D–Supplementary Table 2). After applying the RF method, the top 20 genes were ranked according to mean decrease accuracy (MDA), namely KREMEN2, CCDC142, YTHDF3, EXOC3L2, DSG3, ZNF771, MSANTD1, ZDHHC20, GDF2, OPTC, SOX4, COG3, ZNF497, RASL10A, DPF1, SLC30A3, C2orf70, RASGRP1, PIGK, and SPNS1 (Fig. 5E and. F). Finally, the intersection of genes from LASSO, SVM, and RF analysis yielded six feature genes: MSANTD1, RASL10A, SOX4, YTHDF3, ZNF497, and ZNF771 (Fig. 5G).

### 3.5 Evaluation of feature genes

**Fig. 6:**
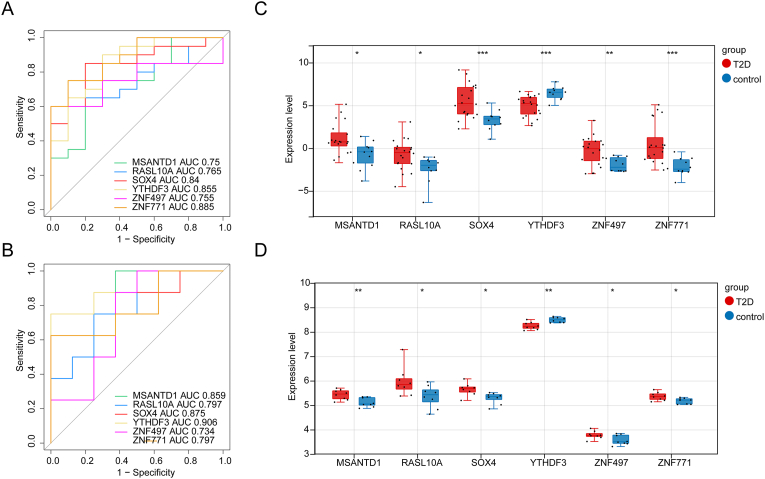
ROC curve and expression analysis of feature genes. (A) Receiver operating characteristic (ROC) curves of six feature genes in GSE153315 dataset. (B) ROC curves of six feature genes in GSE15932 dataset. (C) Expression levels of six feature genes in GSE153315 dataset. (D) Expression levels of six feature genes in GSE15932 dataset. T2D in the diagram represents T2DM. ∗P < 0.05, ∗∗P < 0.01, ∗∗∗P < 0.001.

The area under the curve (AUC) values for all six feature genes exceeded 0.75, demonstrating their excellent ability to distinguish T2DM from normal samples (Fig. 6A). In the GSE15932 dataset, AUC values for all feature genes remained above 0.7, further confirming their strong diagnostic potential (Fig. 6B). A box plot illustrated significant differences in the expression levels of these feature genes in both GSE153315 and GSE15932. Specifically, MSANTD1, RASL10A, SOX4, ZNF497, and ZNF771 were significantly upregulated, while YTHDF3 was notable downregulated in T2DM samples (Fig. 6C and. D).

### 3.6 Single gene functional annotation analysis

**Supplementary Fig. 1:**
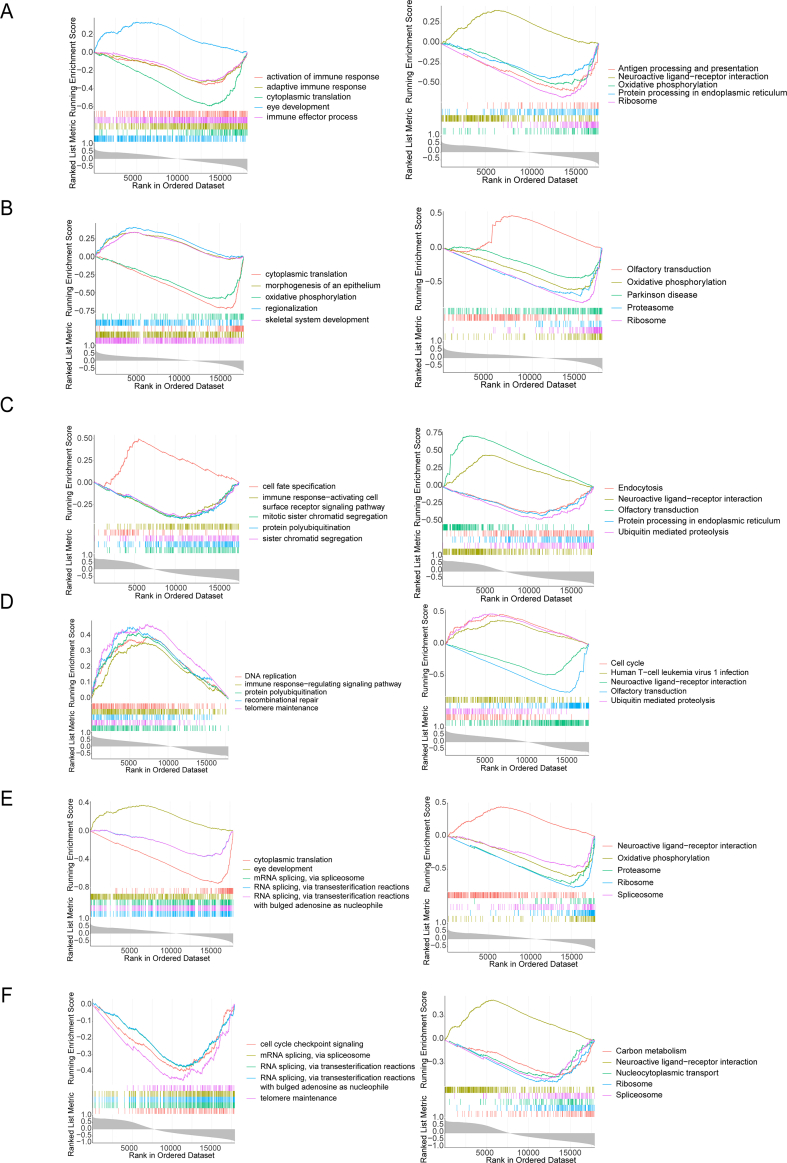
Single gene set enrichment analysis (GSEA) of feature genes. (A) Top five Gene Ontology Biological Process (GO BP) and Kyoto Encyclopedia of Genes and Genomes (KEGG) pathways of MSANTD1. (B) Top five GO BP and KEGG pathways of RASL10A. (C) Top five GO BP and KEGG pathways of SOX4. (D) Top five GO BP and KEGG pathways of YTHDF3. (E) Top five GO BP and KEGG pathways of ZNF497. (F) Top five GO BP and KEGG pathways of ZNF771.

MSANTD1 was involved in GO biological process (BP) pathways, including activated immune response, adaptive immune response, and cytoplasmic translation, and was enriched in KEGG pathways such as antigen processing and presentation, fatty acid metabolism, and oxidative phosphorylation (Supplementary Fig. 1A). RASL10A participated in GO BP pathways such as cytoplasmic translation, epithelial cell morphogenesis, and oxidative phosphorylation, while its KEGG pathways included olfactory transduction, Parkinson's disease, and ribosomes (Supplementary Fig. 1B). The top five GO BP pathways associated with SOX4 included cell fate specification, immune response-activated cell surface receptor signaling, mitotic sister chromatid segregation, and protein polyubiquitination. Additionally, among the top five KEGG enrichment pathways, olfactory transduction and neuroactive ligand-receptor interaction were notably upregulated (Supplementary Fig. 1C). The top five GO BP pathways for YTHDF3 included DNA replication, immune response-regulating signaling, protein polyubiquitination, recombinational repair, and telomere maintenance, while its top KEGG pathways included the cell cycle, olfactory transduction, ubiquitin-mediated proteolysis, and neuroactive ligand-receptor interaction (Supplementary Fig. 1D). ZNF497 was implicated in GO BP pathways such as cytoplasmic translation, eye development, and mRNA splicing via the spliceosome, with KEGG pathways associated with oxidative phosphorylation, the spliceosome, and the proteasome (Supplementary Fig. 1E). Lastly, ZNF771 was enriched in GO BP pathways such as cell cycle checkpoint signaling, RNA splicing via transesterification reactions, and telomere maintenance, and it was involved in KEGG pathways such as carbon metabolism and nucleocytoplasmic transport (Supplementary Fig. 1F). These analyses strongly suggested that the roles of these hub genes in T2DM are closely linked to immune responses.

### 3.7 Identification of DE-lncRNAs and DE-miRNAs

**Supplementary Fig. 2:**
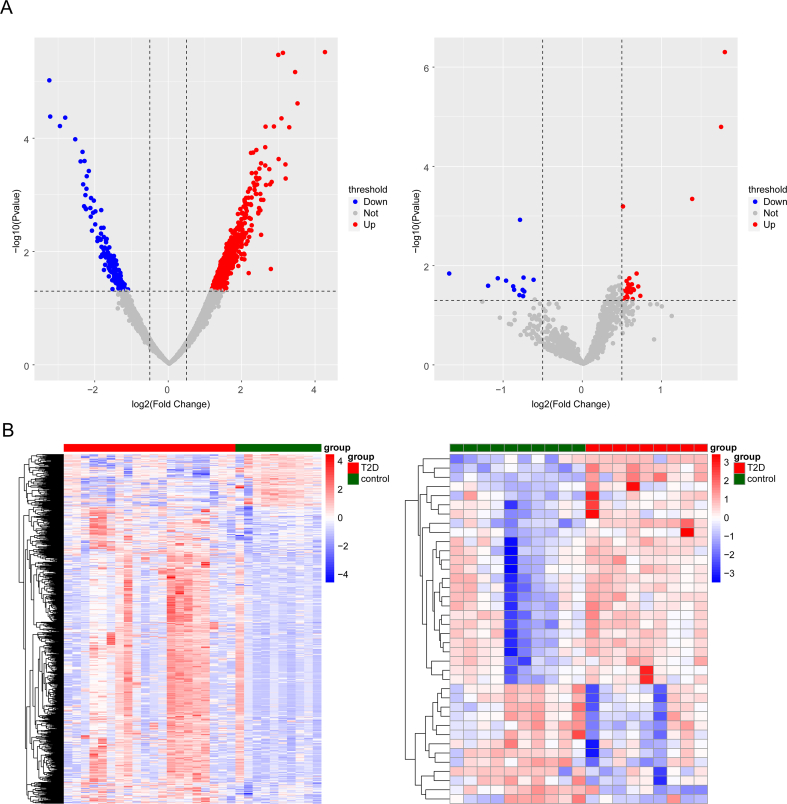
DE-lncRNAs and DE-miRNAs. (A) Volcano map depicting DE-lncRNAs and DE-miRNAs in the GSE153315 and GSE21321 datasets. (B) Heatmap illustrating DE-lncRNAs and DE-miRNAs in the GSE153315 and GSE21321 datasets. T2D in the diagram represents T2DM.

A total of 1012 DE-lncRNAs and 38 DE-miRNAs were identified between T2DM and normal samples. Their expression patterns were visualized using a volcano plot and heat map (Supplementary Fig. 2A, Supplementary Fig. 2B).

### 3.8 Drug prediction for feature genes based on in-silico analysis

**Supplementary Fig. 3:**
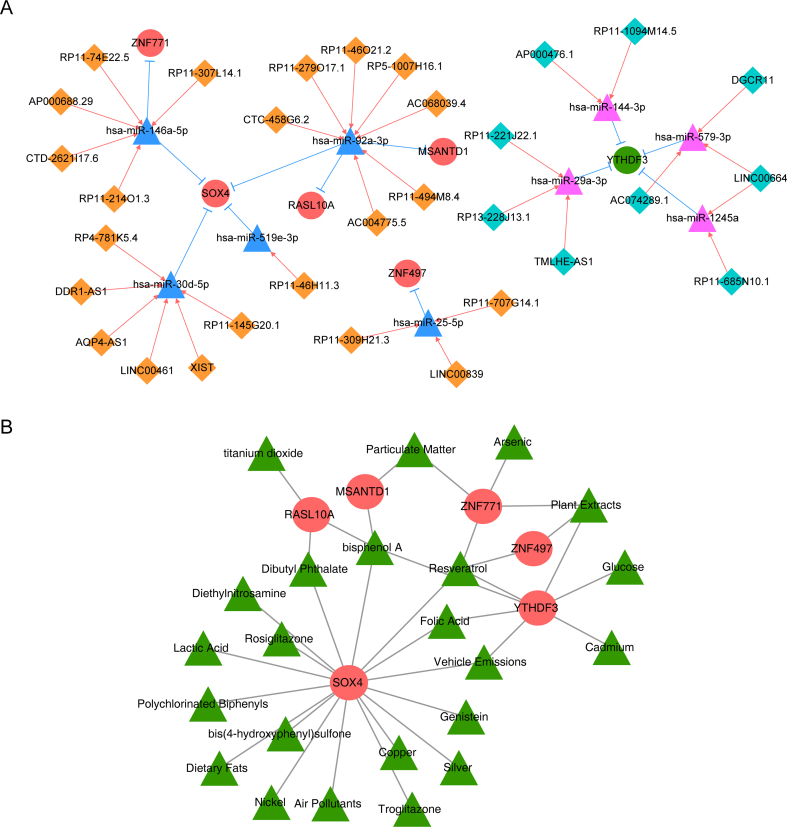
Construction of ceRNA networks and drug prediction**.** (A) The network of mRNA-miRNA-lncRNA. (B) The drug-feature genes network.

Prediction results from miRWalk 3.0 and ENCORI were de-duplicated and merged, yielding 2219 mRNA–miRNA relationship pairs (1414 miRNAs and 6 mRNAs). These predicted miRNAs intersected with DE-miRNAs, resulting in 12 mRNA–miRNA relationship pairs, comprising nine miRNAs and six mRNAs. A total of 51 mRNA–miRNA–lncRNA trios (31 lncRNAs, 9 miRNAs, and 6 mRNAs) were identified, constituting the ceRNA network. Notable interactions included RP11-221J22.1–hsa-miR-29a-3p–YTHDF3, RP11-214O1.3–hsa-miR-146a-5p–SOX4, and RP11-214O1.3–hsa-miR-146a-5p–ZNF771 (Supplementary Fig. 3A). In total, 23 drugs associated with T2DM were identified based on the feature genes. The drug–target network diagram featured interactions such as bisphenol A–MSANTD1, dibutyl phthalate–RASL10A, air pollutants–SOX4, vehicle emissions–YTHDF3, plant extracts–ZNF497, and particulate matter–ZNF771 (Supplementary Fig. 3B).

### 3.9 Feature genes significantly correlated with immune cells

**Fig. 7:**
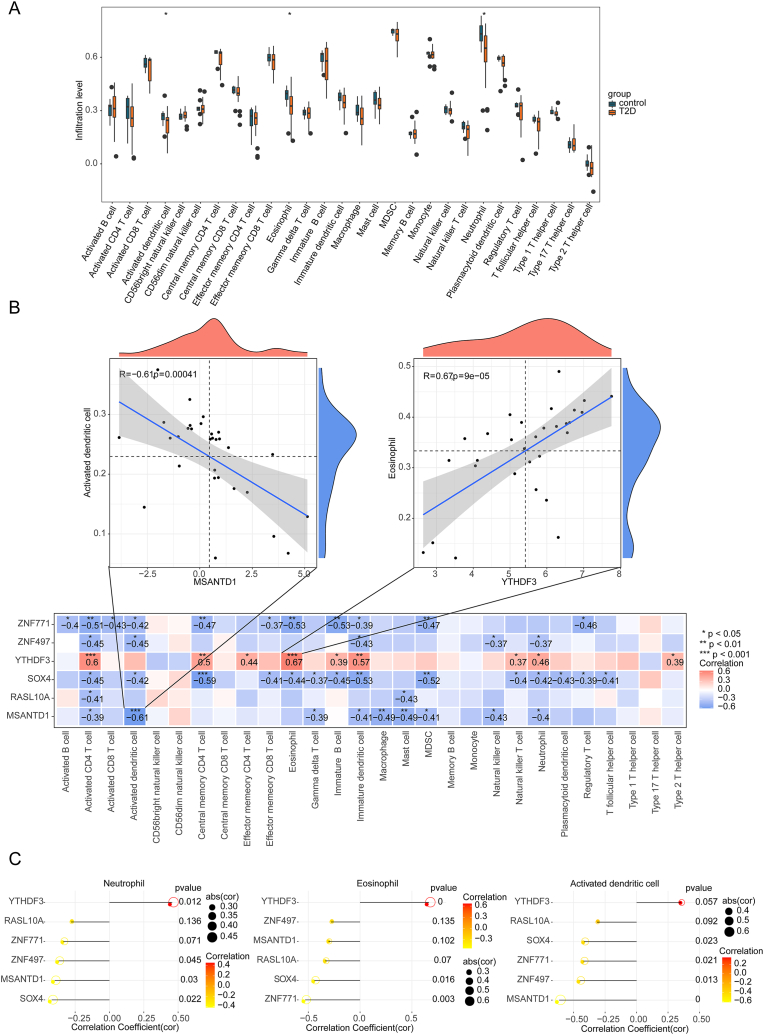
Immune infiltration analysis of feature genes. (A) Immune correlation analysis of 28 types of immune cells from all samples in the GSE153315 dataset. T2D in the diagram represents T2DM. (B) Correlations between feature genes and 28 types of immune cells. (C) Lollipop map of the correlation between feature genes and differential immune cells. ∗P < 0.05.

A box plot revealed significant differences (*P* < 0.05) in the presence of dendritic cells, eosinophils, and neutrophils between T2DM and normal samples (Fig. 7A). Among the feature genes, YTHDF3 exhibited the strongest positive correlation with eosinophils, while MSANTD1 showed the most pronounced negative correlation with activated dendritic cells (Fig. 7B). These correlations between feature genes and differential immune cells were further illustrated using a lollipop plot (Fig. 7C).

### 3.10 Results of wet-lab experiments

**Fig. 8:**
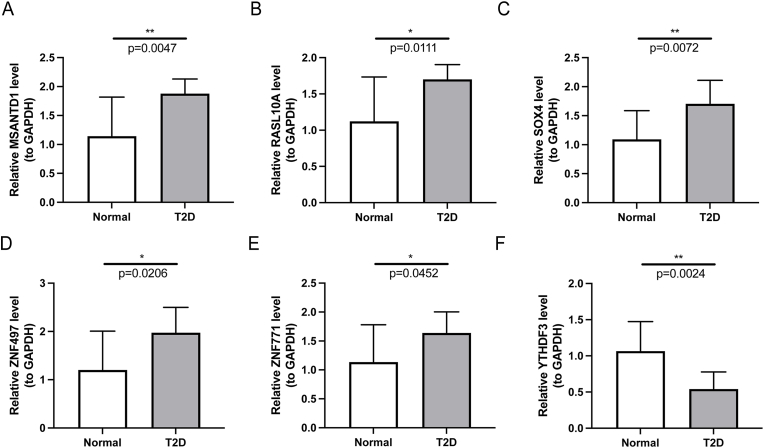
The expression of feature genes between T2DM and normal groups. T2D in the diagram represents T2DM. ∗P < 0.05, ∗∗P < 0.01.

To further validate the findings, the expression levels of six feature genes were examined using qRT-PCR. Compared to the normal group, the expression levels of MSANTD1, RASL10A, SOX4, ZNF497, and ZNF771 were significantly upregulated in the T2DM group, while YTHDF3 was notably downregulated (Fig. 8). The qRT-PCR results were consistent with the gene expression patterns observed in the GSE153315 dataset, reinforcing the reliability of the findings.

## 4 Discussion

This study underscores the potential of fatty acids and FMGs as valuable biomarkers for disease prevention and management, particularly in T2DM. Previous studies have established a link between elevated total plasma-free fatty acids and diabetes risk [[16], [17], [18]]. In this analysis, six feature genes - MSANTD1, RASL10A, SOX4, YTHDF3, ZNF497, and ZNF771 emerged as potential biomarkers for T2DM, identified through three machine learning algorithms: LASSO logistic regression, SVM-RFE, and RF algorithms. The expression trends of these genes were validated to be consistent with the results obtained in the training dataset. ROC analysis further confirmed the diagnostic potential of these six genes, demonstrating that they could effectively distinguish T2DM samples from healthy controls. This finding underscores their value as diagnostic biomarkers. Additionally, activated dendritic cells, eosinophils, and neutrophils between T2DM and normal samples suggest a potential immune involvement in T2DM pathogenesis. Furthermore, a ceRNA network involving 51 lncRNA–miRNA–mRNA trios was constructed, providing insights into the regulatory interactions that may contribute to T2DM development. Based on the identified feature genes, 23 drugs associated with T2DM were screened, offering potential therapeutic targets.

The transcription factor SOX4, which is highly expressed in pancreatic islets, plays a crucial role in regulating pancreatic development and insulin secretion [19,20]. It is a predominant member of the SOX family involved in β-cell specification and differentiation [21,22]. Sox4 regulates β-cell mass by modulating expression of the T2DM susceptibility gene GRK5 [23]. Notably, its expression is maintained in mature islets, and a study by Xu et al. [24] revealed its necessity for adult β-cell replication by directly regulating the cell cycle. Reduced SOX4 expression has been linked to an increased risk of diabetes due to its impairment of normal β-cell replication. Additionally, mutations in SOX4 are associated with a heightened risk of T2DM. Genome-wide association studies have identified single nucleotide polymorphisms in SOX4 regulatory elements located within the genomic region of Cdkal1 [20,25,26]. Furthermore, SOX4 has been recognized as a novel biomarker of T2DM-induced nephropathy. Additional analyses have indicated its involvement in immune, and inflammatory responses during diabetic nephropathy [27]. Validation efforts revealed that SOX4 expression is upregulated in T2DM, which is consistent with existing reports and suggests that it may influence the disease through these mechanisms.

SOX4 also plays a key role in regulating insulin secretion [19,28]. In adult β-cells, SOX4 influences insulin secretion downstream of the KATP channel [28]. Elevated SOX4 expression has been linked to reduced glucose-induced insulin secretion and increased plasma glucose levels. Specifically, SOX4 hinders insulin release into the extracellular space by upregulating syntaxin-binding protein 6, a regulator of exocytosis. Additionally, SOX4 suppresses adipocyte hyperplasia in obesity by promoting the conversion of adipogenic–non adipogenic cells [29]. Yang et al. [30] identified that nuclear paraspeckle assembly transcript 1 mediates the role of SOX4 in regulating the epithelial-mesenchymal transition in diabetic retinopathy by targeting miR-204. In this study, SOX4 was closely linked to the development of T2DM. SOX4-enriched pathways include cell fate specification, immune response-activated cell surface receptor signaling, and neuroactive ligand-receptor interaction. Ding et al. [31] identified neuroactive ligand-receptor interaction as one of the enriched KEGG pathways for altered metabolites in diabetic retinopathy group and proliferative diabetic retinopathy subgroup. This provides a foundation for further investigation into therapeutic targets associated with these metabolites and lipid alterations.

N^6^-methyladenine (m6A) RNA modification is one of the most prevalent epigenetic processes, affecting RNA, regulated by methyltransferases (“writers”), signal transducers (“readers”), and demethylases (“erasers”) [32]. Disturbances in m6A modifications have been demonstrated to disrupt glucose and lipid metabolism, as well as the immune and inflammatory responses, contributing to diseases like diabetes, obesity, and cardiovascular diseases [[33], [34], [35]]. The YT521-B homology (YTH) domain-containing family of proteins (YTHDFs: YTHDF1, YTHDF2, and YTHDF3) serve as cytoplasmic m6A-binding proteins that significantly influence RNA fate [36]. Bioinformatics analysis has identified an m6A-related module, comprising YTHDF3, LINC00667, MYC, and miR-33a-5p, which can distinguish between gestational DM and normal glucose tolerance samples [37]. In this study, YTHDF3 was enriched in signaling pathways related to DNA replication, telomere maintenance, and immune response regulation in T2DM. It also showed the strongest positive correlation with eosinophils. The concept of telomere attrition offers insights into the pathophysiology of T2DM, suggesting that telomeres and related pathways might play a crucial role in the disease's etiology [38]. Integrated bioinformatics analysis has revealed that DEGs in T2DM are enriched in telomere maintenance pathways [39]. However, compared to the normal group, YTHDF3 was significantly downregulated in the T2DM group, consistent with the downregulation of YTHDF3 expression observed in islet samples from T2DM patients [40].

ZNF771, a gene encoding a transcription regulator, is notably expressed in adipose tissue [41]. Although research on ZNF771 remains limited and its precise function is unclear, its significant expression in adipocytes positions it as a promising candidate for further investigation. An epigenome-wide association study on obesity identified three notable loci: cg18181703 (SOCS3), cg04502490 (ZNF771), and cg02988947 (LIMD2). The methylation status of these loci was associated with body mass index percentile and showed correlations with several features of metabolic syndrome, including insulin responsiveness, central obesity, fat deposition, and plasma lipid levels [42]. ZNF771, one of the key feature genes identified in this research, offers valuable insights through bioinformatics analysis serving as a reference for future research focused on its role in metabolic disorders.

lncRNAs and miRNAs, two major non-coding RNAs, play crucial roles in regulating the pathophysiology of T2DM [43,44]. Through ceRNA mechanisms, lncRNAs and mRNAs act by competitively binding to shared miRNA response elements, thereby modulating post-transcriptional processing. In newly diagnosed T2DM among rural Chinese adults, dysregulated lncRNAs, miRNAs, and mRNAs have been identified as important biomarkers of T2DM. The altered interactions among these molecules were shown to influence the regulation of T2DM pathophysiology [45]. A detailed ceRNA network was constructed, consisting of nine lncRNAs, nine mRNAs, and five miRNAs with Rno-miR-10b-5p and Tgfb2 identified as key regulators of endothelial progenitor cell dysfunction in diabetes [46].

T2DM contributes to both microvascular and macrovascular complications, often leading to chronic microvascular injury and dysfunction [2,47]. The regulatory network involving KCNQ1OT1/circ_0020316–miR-92a-2-5p–mitogen-activated protein kinase 3 has been shown to play a role in T2DM-induced vascular injury [48]. In this network, KCNQ1OT1 and circ_0020316 bind to miR-92a-2-5p, which inversely regulates mitogen-activated protein kinase 3. Another lncRNA, Plasmacytoma variant translocation 1 (PVT1), plays a critical role in regulating apoptosis, inflammation, and other processes in various diabetes-related complications [49,50]. PVT1 is significantly upregulated in conditions such as diabetic nephropathy, arthritis, cardiomyopathy, cataracts, and non-alcoholic fatty liver disease [51]. Functioning as a miRNA sponge (binding miR-325-3p, miR-23b-3p, miR-26b, miR-146a, miR-23a-3p, miR-214-3p, and miR-20a-5p), PVT1 modulates downstream protein targets such as Snail, ERG1, WT1, CASP10, MMP2, regulating the occurrence and progression of diabetes-related diseases [[51], [52], [53]]. With advancements in detection and application technology, PVT1 shows potential as a non-invasive biomarker and therapeutic target in the development of new treatments for diabetes.

Moreover, a growing body of clinical research suggests that the abnormal expression of noncoding RNAs can serve as a valuable indicator for the diagnosis, prevention, and treatment of diabetic foot ulcers (DFU). For instance, treatment with miRNA-497 in both in vivo and in vitro settings reduced the expression of proinflammatory cytokines such as interleukin-6, interleukin-1β, and tumor necrosis factor-α, thereby accelerating wound healing in DFU [54]. Similarly, the overexpression of the lncRNA CASC2 enhanced wound healing through the miR-155/hypoxia-inducible factor-1α axis in DFU [55]. Previous studies have also highlighted the critical role of miRNAs in the human pancreatic islets and β-cell function, with miR-192 and miR-146a identified as downregulated in these processes. In this study, we constructed a ceRNA network consisting of 51 lncRNA–miRNA–mRNA pairs (31 lncRNAs, 9 miRNAs, and 6 mRNAs), including significant interactions such as RP11-221J22.1–hsa-miR-29a-3p–YTHDF3, RP11-214O1.3–hsa-miR-146a-5p–SOX4, and RP11-214O1.3–hsa-miR-146a-5p–ZNF771. Notably, miR-29a emerged as a mediator of glucose-induced β-cell dysfunction, and its upregulation in glucose-induced β-cells may contribute to the progression from impaired glucose tolerance to T2DM [56].

The drug-target network results showed that resveratrol was associated with several signature genes (SOX4, YTHDF3, ZNF497 and ZNF771). The study showed that resveratrol supplementation was effective in reducing insulin resistance, leading to a positive effect on glycemic control. It has also shown significant benefits in improving chronic inflammation, reducing oxidative stress and regulating the expression of related miRNAs in diabetic patients [57]. This study also found that bisphenol A was associated with MSANTD1 and dibutyl phthalate was associated with RASL10A. Apoptosis and oxidative stress are the main mechanisms of bis(2-ethylhexyl) phthalate, dibutyl phthalate and bisphenol A mixture induced T2DM. It is worth noting that, probiotic intervention alleviates the redox imbalance in rat pancreas [58].

Recent studies have found that genes involved in fatty acid metabolism contribute to the risk prediction of a variety of diseases, including cancer and cardiovascular disease [59]. The diagnostic model based on FMGs PRKAR2B/ANXA1 has good predictive value for the early and late stages of diabetic nephropathy [60]. However, the diagnostic performance of FMGs for T2DM remains to be explored. The signature genes identified in this study showed a strong association with the risk of T2DM. In terms of therapeutic intervention, by regularly monitoring the changes in the levels of these genes in patients, doctors can more accurately adjust the treatment plan, thereby ensuring the effectiveness of treatment and patient safety. However, the application of these genes in clinical practice still faces a series of challenges, including stability issues, accuracy of detection methods, and so on. Therefore, more studies are needed to explore whether these genes can be used as biomarkers in clinical practice. For example [1]: Equipment, technology and processes need to be optimized [2]. Prospective randomized controlled trials are needed.

Although this study provides valuable insights into disease mechanisms and biomarkers, several limitations remain. Due to the complexity of biological systems, the completeness of data, and the power of algorithms, the results of bioinformatics analysis may not be comprehensive enough. In addition, physiological and pathological processes involve the interaction of multiple tissues and organs, and effective targets at the cellular or molecular level may be influenced by multiple factors. To overcome these limitations, it is necessary to verify the function and molecular mechanism of the predicted targets by in vivo experiments or animal models, improve study design, expand the sample size of clinical trials to evaluate its sensitivity and specificity, and explore its feasibility as a T2DM biomarker.

## 5 Conclusions

In conclusion, six feature genes were identified, which with AUC >0.70 showed excellent diagnostic value for T2DM, and thus were considered as hub genes of T2DM, including MSANTD1, RASL10A, SOX4, YTHDF3, ZNF497, and ZNF771. The investigation into the molecular mechanisms of these genes, conducted through bioinformatics analysis, provides a foundation for T2DM-related studies. The findings not only contribute valuable insights into the roles of MSANTD1, RASL10A, SOX4, YTHDF3, ZNF497, and ZNF771 in T2DM but also offer new directions and potential drug targets for the treatment of the disease. Future research will continue to explore the specific roles of these feature genes related to fatty acid metabolism in the development of T2DM.

## Informed consent

Written informed consent was obtained from individual or guardian participants.

**Table:** Glossary

**Abbreviations**	**Full name**
T2DM	Type 2 diabetes mellitus
FMGs	Fatty acid metabolism-related genes
WGCNA	Weighted Gene Co-Expression Network Analysis
DM	Diabetes mellitus
GEO	Gene Expression Omnibus database
MSigDB	Molecular Signatures Database
GSEA	Gene set enrichment analysis
lncRNA	Long non-coding RNA
miRNA	MicroRNA
DEGs	Differentially expressed genes
FAM	score Fatty acid metabolism score
ssGSEA	Single-sample Gene Set Enrichment Analysis
LASSO	Least absolute shrinkage and selection operator
SVMs	Support vector machines
RFE	Recursive feature elimination
RF	Random forest
ROC	Receiver operating characteristic
GO	Gene Ontology
KEGG	Kyoto Encyclopedia of Genes and Genomes analyses

## Author contributions

**Ji Yang**: Conceptualization. **Ji Yang and Yikun Zhou**: methodology. **Ji Yang, Jiarui Zhang and Yongqin Zheng**: software. **Yikun Zhou and Jundong He**: validation. **Ji Yang and Jundong He**: formal analysis. **Ji Yang and Yikun Zhou**: investigation. **Jundong He**: resources. **Ji Yang and Jiarui Zhang**: data curation. **Ji Yang and Yikun Zhou**: writing - original draft preparation. **Ji Yang, Yikun Zhou and Jundong He**: writing—review and editing. **Ji Yang and Yongqin Zheng**: visualization. **Yikun Zhou and Jundong He**: supervision. **Jundong He**: project administration. **Jundong He**: funding acquisition. All authors have read and agreed to the published version of the manuscript.

## Data sharing statement

The datasets (GSE153315, GSE21321, and GSE15932) analyzed in this study were downloaded from the Gene Expression Omnibus database (https://www.ncbi.nlm.nih.gov/).

## Ethical disclosure

The authors state that this study was approved by the Ethics Committee of The First People's Hospital of Yunnan Province (NO: KHLL2021-KY012). Written informed consent was obtained from individual or guardian participants.

Registry and the Registration No. of the study/trial: KHLL2021-KY012.

Approval date of Registry and the Registration No. of the study/trial: January 26, 2021.

## Approval of the research protocol

Approval was granted by the ethics committee of The First People's Hospital of Yunnan Province.

## Funding sources

This work was supported by the Yunnan Province “Prosper Yunnan Talent Support Program” [YNWR-QNBJ-2018-070], Reserve talents of young and middle-aged academic and technical leaders in Yunnan Province [2018HB050], National Clinical Key Specialty Cultivation Project Platform for Endocrinology [2024NMKFKT-01].

## Declaration of competing interest

The authors declare that they have no known competing financial interests or personal relationships that could have appeared to influence the work reported in this paper.

## Data Availability

The datasets (GSE153315, GSE21321, and GSE15932) analyzed in this study were downloaded from the Gene Expression Omnibus database (https://www.ncbi.nlm.nih.gov/).
